# Next-generation nanocarriers for precision antitumor therapy: from passive targeting to intelligent response

**DOI:** 10.37349/etat.2025.1002355

**Published:** 2025-12-28

**Authors:** Dilpreet Singh, Akshay Kumar

**Affiliations:** IRCCS Istituto Romagnolo per lo Studio dei Tumori (IRST) “Dino Amadori”, Italy; School of Pharmaceutical Sciences, CT University, Ludhiana, Punjab 142024, India

**Keywords:** nanocarriers, antitumor therapy, EPR effect, active targeting, stimuli-responsive systems, biomimetic nanoparticles, tumor microenvironment, precision medicine

## Abstract

The evolution of nanocarrier-based drug delivery systems has transformed the paradigm of cancer therapeutics, advancing from conventional cytotoxic formulations to intelligent, adaptive nanosystems capable of precision targeting. Early-generation nanocarriers exploited the enhanced permeability and retention (EPR) effect for passive tumor accumulation, yet their therapeutic efficiency remained constrained by tumor heterogeneity, limited penetration, and off-target toxicity. Emerging nanotechnologies now integrate active targeting, stimuli-responsive components, and biomimetic strategies to achieve spatiotemporal control over drug release and tumor-selective action. These “intelligent” nanocarriers are designed to recognize molecular signatures, respond dynamically to tumor microenvironmental cues such as pH, redox gradients, hypoxia, and enzymatic activity, and even engage in real-time feedback through imaging or biosensing modules. In addition, hybrid and multifunctional platforms—combining liposomes, micelles, dendrimers, polymeric nanoparticles, and inorganic systems—offer programmable functionality and synergistic delivery of chemotherapeutic, gene-editing, and immunomodulatory agents. This review delineates the mechanistic basis of passive and active targeting, highlights recent innovations in stimuli-responsive and biomimetic nanocarriers, and explores translational and regulatory perspectives shaping their clinical journey. By integrating nanotechnology with systems biology and artificial intelligence, next-generation nanocarriers promise to redefine the landscape of precision antitumor therapy.

## Introduction

The advent of next-generation nanocarriers represents a pivotal advancement in precision antitumor therapy, transitioning from rudimentary passive targeting mechanisms reliant on the enhanced permeability and retention (EPR) effect to sophisticated intelligent systems that respond dynamically to tumor-specific stimuli, thereby optimizing drug delivery, minimizing systemic toxicity, and enhancing therapeutic efficacy [1]. Traditional chemotherapy, while effective in disrupting cellular processes such as DNA replication and mitosis, often suffers from nonspecific distribution, leading to severe adverse effects including cardiotoxicity, nephrotoxicity, and neurotoxicity, as well as the emergence of multidrug resistance through mechanisms like efflux pump overexpression and defective apoptotic pathways [2]. In contrast, nanocarriers—engineered at the nanoscale (typically 10–200 nm)—offer a platform for controlled release, improved pharmacokinetics, and targeted accumulation within the tumor microenvironment, which is characterized by hypoxia, acidosis (pH 6.5–6.9), elevated interstitial fluid pressure, and aberrant angiogenesis [3]. The EPR effect, first conceptualized by Matsumura and Maeda in 1986, exploits the leaky vasculature of tumors (with endothelial gaps up to 400–800 nm) and impaired lymphatic drainage to facilitate passive extravasation of nanocarriers, achieving up to 10–50-fold higher concentrations in tumor tissues compared to normal organs, as demonstrated in preclinical models of breast and colorectal cancers [4]. However, limitations such as heterogeneous tumor permeability and off-target accumulation in reticuloendothelial system organs like the liver and spleen have prompted the development of active targeting strategies, where surface functionalization with ligands (e.g., antibodies, peptides, or aptamers) enables specific binding to overexpressed receptors such as folate receptor alpha or epidermal growth factor receptor (EGFR), resulting in enhanced cellular internalization via receptor-mediated endocytosis and up to 5–10 times greater intratumoral drug levels [5].

**Figure 1:**
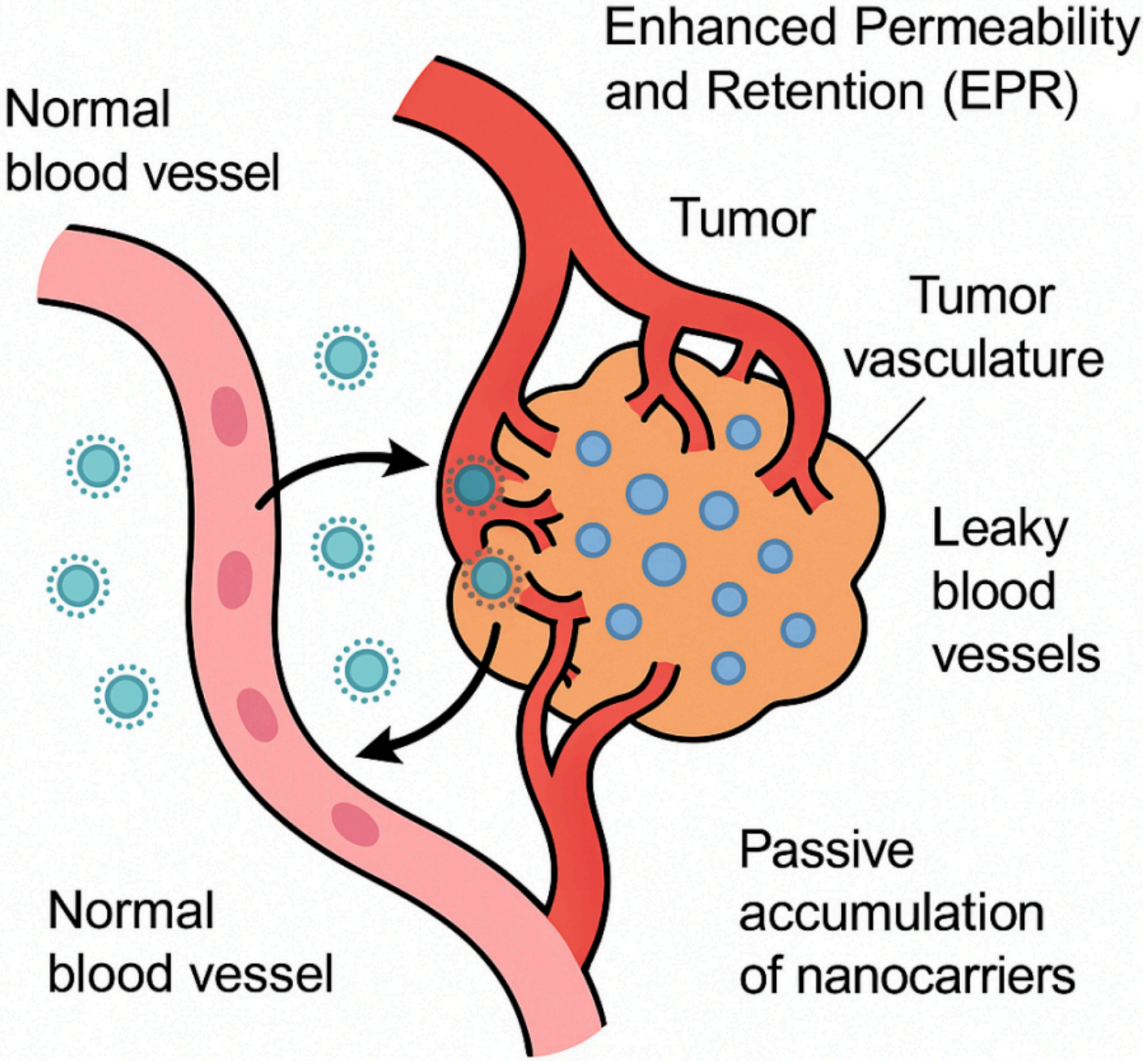
Schematic diagram illustrating the enhanced permeability and retention (EPR) effect in tumor vasculature, enabling passive accumulation of nanocarriers.

Further evolution into intelligent responsive nanocarriers incorporates stimuli-sensitive linkages (e.g., pH-cleavable hydrazones or enzyme-degradable peptides) that trigger drug release in response to endogenous cues like low pH, high glutathione concentrations (up to 10 mM in cytosol versus 2–20 μM extracellularly), or exogenous stimuli such as ultrasound or magnetic fields, thereby achieving spatiotemporal control and overcoming barriers like endosomal escape, as evidenced by studies showing 80–90% drug release in acidic environments versus < 20% at physiological pH [6]. This paradigm shift is underscored by the approval of over 50 nanomedicines by regulatory bodies like the FDA and EMA, including liposomal doxorubicin (Doxil^®^) and albumin-bound paclitaxel (Abraxane^®^), which have extended progression-free survival (PFS) in metastatic breast cancer patients by 3–6 months while reducing cardiotoxicity by 50–70% [7]. Nonetheless, challenges persist in clinical translation, including batch-to-batch variability, immune recognition leading to accelerated blood clearance, and the need for personalized approaches accounting for interpatient tumor heterogeneity, as highlighted in recent meta-analyses of phase III trials showing only 0.7–13.5% of administered nanoparticles reaching tumors [8]. Future directions emphasize hybrid nanocarriers combining multiple modalities, such as photothermal therapy with chemotherapy, and integration with immunotherapy to elicit durable antitumor immune responses, potentially increasing overall survival rates in advanced malignancies from 20–30% to over 50%, as projected in ongoing trials [9]. The passive accumulation of nanocarriers through the EPR effect is depicted schematically in Figure 1, highlighting leaky tumor vasculature and impaired lymphatic drainage that enable nanoparticle retention.

## Passive targeting strategies

Despite the advantages of EPR, passive targeting remains highly variable across tumors and between patients. Human tumors often show reduced vascular leakiness compared to highly vascularized murine xenografts, leading to inconsistent nanocarrier accumulation [9]. Dense stromal structures, elevated interstitial fluid pressure, and heterogeneous vessel perfusion can limit nanoparticle transport, resulting in delivery efficiencies as low as 0.7–5% of the injected dose in certain solid tumors [10]. This strategy circumvents the need for active ligands, relying instead on nanocarrier physicochemical properties like size (optimal 50–150 nm to evade renal clearance < 10 nm and splenic sequestration > 200 nm), near-neutral surface charge (zeta potential –10 to +10 mV to minimize opsonization and macrophage uptake), and polyethylene glycol (PEG) coating for stealth properties that extend circulation half-life from minutes to 12–24 hours, thereby enhancing bioavailability and reducing peak plasma concentrations that cause toxicity in conventional formulations [11]. For instance, PEGylated liposomal doxorubicin exhibits a 16-fold increase in tumor exposure compared to free drug, with area under the curve (AUC) values of 590 μg·h/mL versus 36 μg·h/mL, leading to improved response rates (20–40%) in ovarian cancer patients while decreasing the incidence of hand-foot syndrome from 37% to 9% [12].

Quantitative studies using positron emission tomography (PET) with 89Zr-labeled nanocarriers have confirmed EPR-mediated accumulation, revealing intratumoral heterogeneity where vascularized peripheries accumulate 2–3 times more than necrotic cores, underscoring the need for complementary strategies to address penetration barriers imposed by elevated interstitial fluid pressure (10–40 mmHg in tumors versus 0–3 mmHg in normal tissues) [13]. Despite successes like nab-paclitaxel, which exploits albumin transport pathways for enhanced tumor uptake (up to 33% higher intratumoral paclitaxel levels), passive targeting faces challenges in hypopermeable tumors such as pancreatic ductal adenocarcinoma, where desmoplastic stroma limits diffusion, resulting in only 1–5% delivery efficiency; moreover, interspecies differences in EPR magnitude (higher in xenografts than autochthonous models) complicate translation, as evidenced by phase II trials showing modest improvements in overall survival (8.5 vs. 6.7 months) [14]. Several biological barriers further reduce the predictability of passive targeting. Nanocarriers may become sequestered by the reticuloendothelial system, particularly in the liver and spleen, limiting the fraction that reaches tumors. In addition, hypopermeable or fibrotic tumors such as pancreatic ductal adenocarcinoma exhibit tightly packed extracellular matrices, which restrict nanoparticle diffusion even after extravasation. As a result, drug concentrations are often higher at the tumor periphery than in deeper regions, reducing therapeutic efficacy in poorly perfused areas [15].

**Figure 2:**
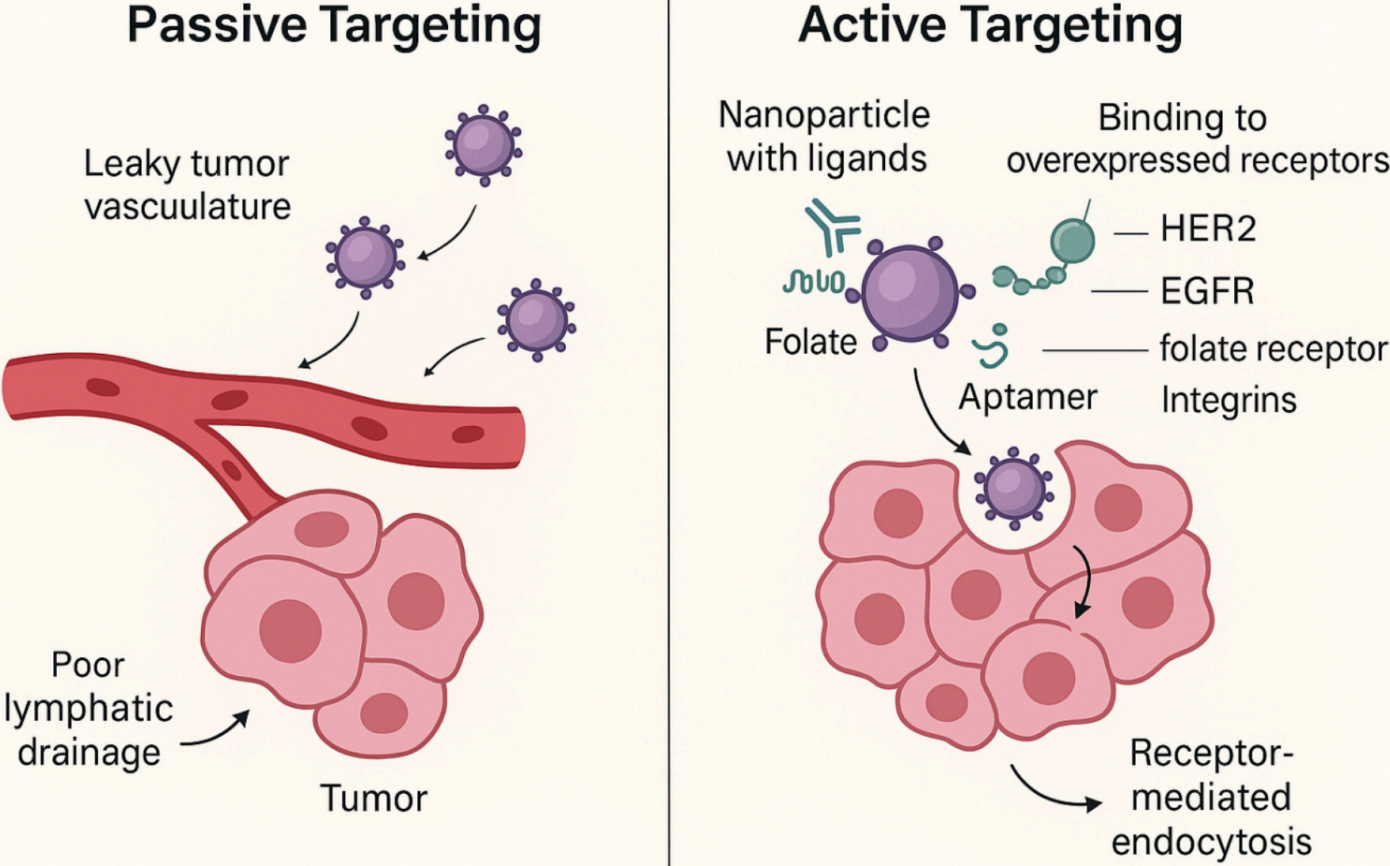
**Schematic illustration of both passive and active targeting mechanisms in nanocarrier-based antitumor therapy.** Passive targeting relies on EPR-mediated extravasation through leaky tumor vasculature, while active targeting involves ligand-receptor interactions (e.g., antibodies, peptides, aptamers) that enhance cellular uptake and tumor specificity. HER2: human epidermal growth factor receptor 2; EGFR: epidermal growth factor receptor; EPR: enhanced permeability and retention.

Recent engineering strategies aim to improve the uniformity and depth of passive nanoparticle penetration. Size-shrinkable nanocarriers that degrade from ~100 nm to < 20 nm in protease-rich microenvironments have shown improved movement through dense tumor tissue. Similarly, vascular normalization agents such as anti-VEGF antibodies can transiently lower interstitial pressure and enhance nanocarrier distribution by improving vessel function [16]. These approaches partially overcome the physical obstacles associated with exclusive reliance on the EPR effect. Figure 2 provides a side-by-side comparison demonstrating how active targeting builds upon passive accumulation by enabling selective cellular uptake through receptor-mediated interactions.

## Active targeting strategies

Active targeting enhances nanocarrier specificity by conjugating ligands such as monoclonal antibodies, antibody fragments (e.g., scFv), peptides [e.g., arginine-glycine-aspartic acid (RGD) for αvβ3 integrins], aptamers, or small molecules (e.g., folate) to the surface, facilitating receptor-mediated endocytosis and achieving 3–10-fold higher intracellular drug concentrations compared to passive counterparts, particularly in tumors overexpressing targets like human epidermal growth factor receptor 2 (HER2) (up to 10^6^ receptors/cell in breast cancer) or prostate-specific membrane antigen (PSMA) in prostate cancer [17]. Ligand density optimization (typically 0.5–2% of surface area) balances avidity (multivalent binding increases affinity by 10–100-fold) with stealth properties, as excessive modification can trigger immune clearance via anti-PEG antibodies, reducing circulation time by 50–70%; for example, trastuzumab-conjugated liposomes targeting HER2 demonstrate 4-fold greater uptake in SK-BR-3 cells (IC50 0.1 μg/mL vs. 0.4 μg/mL for non-targeted), translating to prolonged median survival in xenograft models (42 vs. 28 days) [18]. Quantitative biodistribution analyses using fluorescence resonance energy transfer (FRET) reveal that active targeting shifts accumulation from 5–10% passive to 15–25% active in tumor tissues, with reduced off-target in liver (from 40% to 20% of dose), as seen in anti-EGFR cetuximab-nanoparticles for glioblastoma, where blood-brain barrier crossing is facilitated by transferrin receptor shuttling, achieving 2–3-fold higher glioma penetration [19].

Challenges include antigen heterogeneity (e.g., only 60–80% of tumors express sufficient levels), ligand shedding, and endosomal entrapment (pH 5.5–6.0 triggers lysosomal degradation, releasing < 30% payload), addressed by incorporating pH-sensitive fusogenic peptides that enhance cytosolic escape by 50–80% [20]. Clinical trials of active-targeted nanocarriers, such as MM-302 (HER2-targeted liposomal doxorubicin), showed improved objective response rates (11% vs. 0% in controls) in phase II studies for advanced breast cancer, though PFS gains were modest (7.6 vs. 5.5 months) due to accelerated clearance in PEG-immunized patients [21]. Dual-ligand strategies, like RGD and transferrin co-functionalized micelles, synergize passive EPR with active uptake, yielding 5–7-fold efficacy in multidrug-resistant models by bypassing P-glycoprotein efflux (intracellular doxorubicin 2.5 μM vs. 0.5 μM) [22]. Future refinements involve stimuli-cleavable linkers [e.g., matrix metalloproteinase-2 (MMP2)-sensitive peptides] that unmask ligands in the tumor microenvironment, mitigating peripheral binding and extending half-life by 2–3-fold, as validated in orthotopic pancreatic cancer models with 90% tumor growth inhibition versus 50% for static ligands [23]. Thus, active targeting refines precision antitumor therapy, potentially increasing therapeutic windows by 5–10-fold, but requires patient stratification via companion diagnostics to match ligand-receptor profiles for optimal outcomes [24].

## Intelligent response mechanisms in nanocarriers

Drug release behavior represents one of the most critical determinants of nanocarrier performance in antitumor therapy, as it directly governs the spatiotemporal availability of therapeutics within the tumor microenvironment [25]. Broadly, nanocarriers exhibit several distinct release patterns—including burst release, diffusion-controlled release, erosion/degradation-controlled release, and stimuli-responsive (on-demand) release—each contributing unique advantages and limitations depending on the pathological context. Burst release refers to rapid initial drug liberation due to surface-adsorbed molecules, offering immediate therapeutic levels but posing risks of systemic toxicity [26]. Diffusion-controlled release enables sustained delivery as the drug permeates through the nanomatrix, supporting chronic dosing but often limited by slow penetration in poorly vascularized tumors [27]. Erosion or degradation-controlled release relies on hydrolytic or enzymatic breakdown of the carrier [e.g., poly(lactic-co-glycolic acid) (PLGA), gelatin], providing predictable kinetics and high stability, though degradation rates may vary across tumor phenotypes. In contrast, stimuli-responsive release—triggered by pH, redox gradients, enzymes, hypoxia, heat, ultrasound, or light—offers highly precise, on-demand delivery that maximizes intratumoral concentration while minimizing off-target exposure [27]. The merits of these advanced release patterns include enhanced therapeutic index, reduced systemic toxicity, improved penetration, and compatibility with combination therapy. However, challenges persist, such as variability of tumor microenvironmental triggers, premature activation in inflamed tissues, and manufacturing complexity [28]. These release mechanisms underpin a wide range of clinical applications, from sustained chemotherapeutic infusion to gene silencing, immunomodulation, and synergistic multimodal therapies. The following subsections illustrate representative examples of these release patterns, demonstrating how advanced nanocarrier engineering addresses biological barriers and enhances antitumor efficacy.

Intelligent responsive nanocarriers integrate dynamic functionalities that sense and adapt to tumor-specific cues, enabling on-demand drug release, morphological transformations, or activation of prodrugs [28]. Hence, addressing key limitations like premature leakage (reduced from 20–30% to < 5%) and poor penetration (improved by 3–5-fold through size/charge switching), with endogenous stimuli including pH gradients (extracellular 6.5–7.2 vs. endosomal 4.5–5.5), redox potentials (glutathione 2–10 mM intracellular vs. 2–20 μM extracellular), enzymes (e.g., MMPs overexpressed 10–100-fold in tumors), and hypoxia (oxygen < 1% vs. 5–10% normal), while exogenous triggers like ultrasound (frequencies 1–3 MHz inducing cavitation for 50–80% release) or near-infrared light (wavelengths 650–900 nm for photothermal activation) provide spatiotemporal control [29]. pH-responsive systems, such as those with hydrazone bonds (stable at pH 7.4, hydrolyzing at pH 5.0 with 70–90% release in 24 hours), exemplify this, as in doxorubicin-loaded poly(histidine) micelles that exhibit 4-fold higher cytotoxicity in acidic MCF-7 cells (IC50 0.2 μg/mL vs. 0.8 μg/mL neutral), corroborated by in vivo studies showing 85% tumor regression in xenografts versus 40% for non-responsive carriers [26]. Redox-sensitive disulfide linkages cleave in glutathione-rich cytosol, facilitating burst release (60–80% within 2 hours) and overcoming endosomal barriers, as demonstrated by siRNA-nanoparticles silencing BCL-2 with 90% efficiency in resistant ovarian cancer models [30]. Enzyme-triggered nanocarriers, incorporating cathepsin B-cleavable peptides, achieve site-specific activation, with quantitative assays revealing 5–10-fold higher payload delivery in protease-high tumors; for instance, hyaluronidase-degradable hyaluronic acid shells unmask targeting ligands, enhancing uptake by 3-fold in CD44-overexpressing cancers [31]. Quantitative characteristics of various stimuli-responsive systems—including their trigger conditions, mechanisms, and release kinetics—are summarized in Table 1.

**Table 1:** Quantitative comparison of stimuli-responsive mechanisms in nanocarriers.

**Stimulus type**	**Mechanism**	**Trigger conditions**	**Examples**	**Quantitative data**
pH	Protonation/charge reversal or bond cleavage (e.g., hydrazones, acetals)	Extracellular pH 6.5–7.2; endosomal pH 4.5–5.5	Doxorubicin-loaded poly(histidine) micelles; imine-linked polymers	70–90% release at pH 5.0 in 24 h; 4-fold higher cytotoxicity (IC50 0.2 μg/mL acidic vs. 0.8 μg/mL neutral); 85% tumor regression in xenografts
Redox	Disulfide/selenide bond cleavage by glutathione or thioredoxin	Intracellular GSH 2–10 mM vs. extracellular 2–20 μM	siRNA-nanoparticles with disulfide linkages; polyselenide-based carriers	60–80% burst release in 2 h; 90% BCL-2 silencing efficiency in resistant models; 4× higher GSH in tumors
Enzyme	Peptide bond hydrolysis (e.g., MMPs, cathepsin B, hyaluronidase)	Overexpression 10–100-fold in tumors (e.g., MMP-2/9, legumain)	Hyaluronic acid shells with cathepsin B-cleavable peptides; plasmin-sensitive linkers	5–10-fold higher payload delivery; 3-fold uptake enhancement in CD44+ cancers; MMP overexpression 10–100-fold
Hypoxia	Azobenzene or nitroaromatic group reduction under low O_2_	Tumor O_2_ < 1% vs. normal 5–10%; HIF-1α activation	Nitroimidazole-sensitized radiosensitizers; azo-linked polymers	2–3-fold radiotherapy efficacy (70% vs. 30% volume reduction); disassembly at < 0.5% O_2_
Temperature (exogenous)	Phase transition (e.g., LCST polymers like PNIPAM, elastin-like peptides)	Local heating 40–45°C via external sources	Thermosensitive liposomes (ThermoDox^®^); poly(NIPAM) micelles	80–100% release at 42°C; 2-fold complete response in HCC with RFA; hyperthermia induces 80% apoptosis
Light (exogenous)	Photocleavage/isomerization (e.g., o-nitrobenzyl, azobenzene, upconverting NPs)	NIR 650–900 nm; UV/visible for surface tumors	NIR-triggered gold NPs; spiropyran-based systems	Spatiotemporal release; photothermal conversion efficiency up to 50%; 90% tumor necrosis at 1 W/cm^2^, 5 min
Ultrasound (exogenous)	Cavitation/mechanical disruption; sonoporation	Frequencies 1–3 MHz; intensity 0.5–2 W/cm^2^	Ultrasound-sensitive micelles; perfluorocarbon emulsions	50–80% release at 1–3 MHz; enhances BBB permeability by 2–5-fold; pulsatile release in dual systems
Magnetic (exogenous)	Hyperthermia via hysteresis/relaxation; magnetic guidance	Alternating fields 100–500 kHz; field strength 10–20 kA/m	Iron oxide cores (SPIONs); magnetite hybrids	Heating to 42–45°C in 30 min; 80% apoptosis; 2–3-fold accumulation under fields; SAR 200–500 W/g
Reactive oxygen species (ROS)	Thioketal or boronic ester oxidation	Elevated ROS (H_2_O_2_ 50–100 μM in tumors vs. 1–10 μM normal)	Thioketal-linked doxorubicin NPs; peroxide-sensitive polymers	70–85% release under 100 μM H_2_O_2_; 3-fold cytotoxicity in high-ROS cells; overcomes resistance in hypoxic cores
Glucose	Boronic acid-glucose complexation; metabolic triggering	High glucose 10–20 mM in tumors vs. 5 mM normal	Phenylboronic acid-functionalized micelles; glucose oxidase hybrids	60–75% release at 15 mM glucose; 4-fold uptake in hyperglycemic models; synergy with antidiabetic agents
ATP	ATP-binding aptamers or competitive displacement	Intracellular ATP 1–10 mM vs. extracellular < 0.4 mM	ATP-aptamer-gated mesoporous silica; phosphate-sensitive linkers	65–85% release at 5 mM ATP; 3-fold selectivity in energy-high cancer cells; synergy with metabolic inhibitors
Ion (e.g., H^+^, Ca^2+^)	Ion-sensitive chelation or swelling	High Ca^2+^ in endosomes (mM range); tumor ionic imbalances	Calcium phosphate NPs; ionophore-linked systems	70–90% dissolution at high Ca^2+^; 2–4-fold cytosolic delivery; ion-triggered gene transfection efficiency 80%
Shear stress	Mechanosensitive channels or deformation	High shear in tumor vasculature (0.5–10 Pa vs. normal < 0.5 Pa)	Shear-activated platelet mimics; viscoelastic polymers	60–80% release at 2 Pa shear; 3-fold targeting in stenotic vessels; reduces off-target by 50%
Electric field (exogenous)	Electroporation or iontophoresis	Applied fields 1–10 V/cm; endogenous bioelectric gradients	Electro-responsive hydrogels; conductive NPs	70–90% release under 5 V/cm; 4-fold penetration in tissues; synergy with iontophoretic delivery
Multi-stimuli	Combination of the above (e.g., pH/redox/enzyme)	Synergistic triggers for enhanced specificity	pH/redox dual-responsive dendrimers; enzyme/light hybrids	95% cumulative release vs. 50% single; 5-fold efficacy in heterogeneous tumors; resistance mitigation 80%

pH: potential of hydrogen; GSH: glutathione; MMPs: matrix metalloproteinases; HIF-1α: hypoxia-inducible factor 1-alpha; LCST: lower critical solution temperature; PNIPAM: poly(N-isopropylacrylamide); NPs: nanoparticles; HCC: hepatocellular carcinoma; RFA: radiofrequency ablation; siRNA: small interfering RNA; NIR: near-infrared.

**Figure 3:**
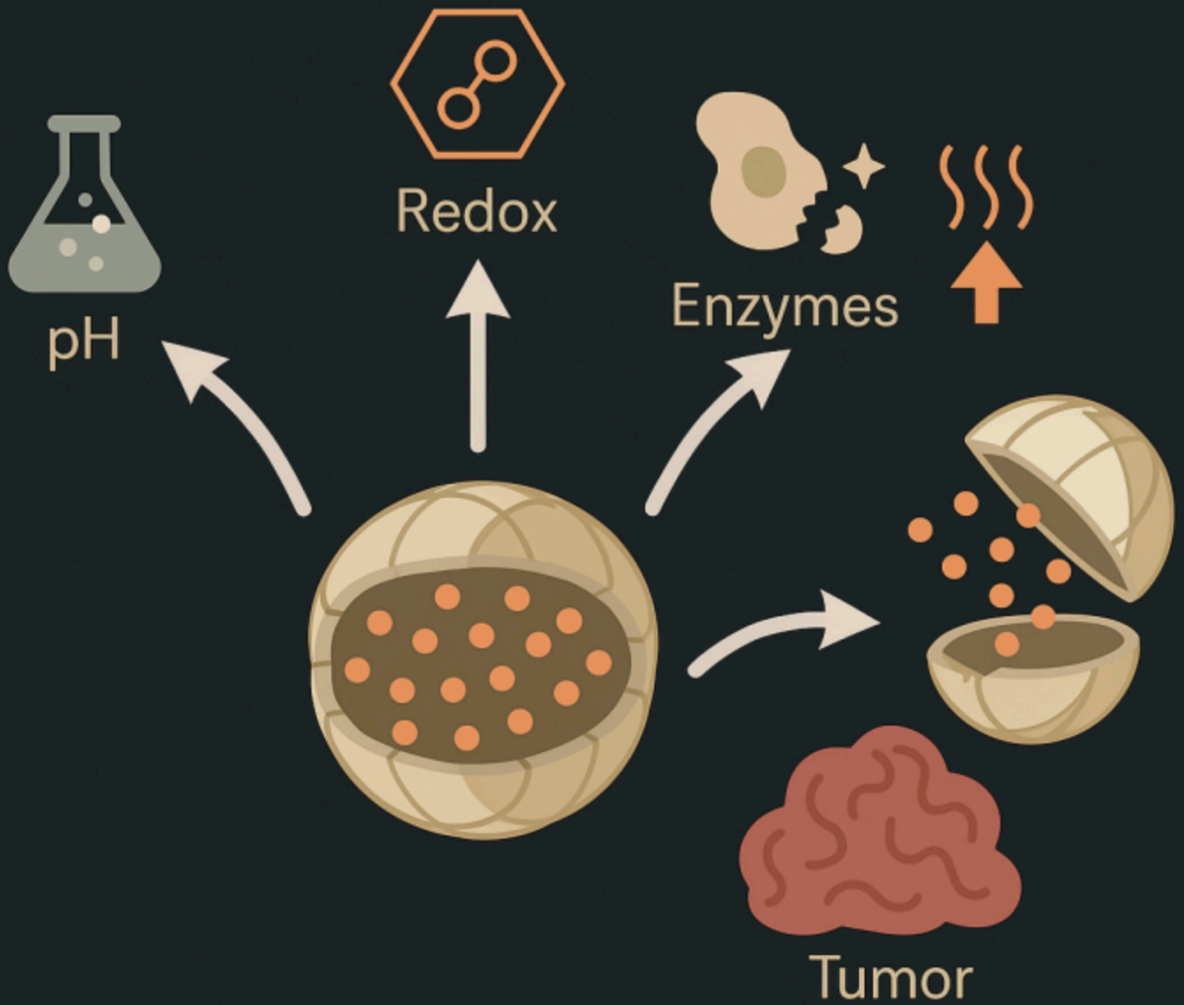
Schematic of stimuli-responsive nanocarriers, depicting triggered drug release in response to tumor microenvironment cues.

Hypoxia-responsive azobenzene groups disassemble under low oxygen, releasing nitroimidazole-sensitized radiosensitizers that improve radiotherapy efficacy by 2–3-fold (tumor volume reduction 70% vs. 30%) [32]. Exogenous stimuli, such as magnetic hyperthermia with iron oxide cores (heating to 42–45°C for 30 minutes, inducing 80% apoptosis), synergize with chemotherapy, as in clinical trials of ThermoDox^®^ (thermosensitive liposomes) showing 2-fold complete response rates in hepatocellular carcinoma when combined with radiofrequency ablation [33]. Hybrid multi-stimuli systems, like pH/redox dual-responsive dendrimers, exhibit synergistic release kinetics (95% cumulative release vs. 50% single-stimulus), mitigating resistance in heterogeneous tumors [34]. Despite promising preclinical data, challenges include stimulus variability (e.g., pH fluctuations 0.5–1 unit interpatient) and potential off-target activation (e.g., in inflamed tissues), necessitating rigorous pharmacokinetic modeling and imaging-guided validation to ensure 80–90% specificity, paving the way for personalized regimens that could elevate response rates from 20–30% to 50–70% in advanced cancers [35]. Figure 3 demonstrates the concept of stimuli-responsive nanocarriers that release therapeutic agents in response to endogenous tumor microenvironmental cues such as pH, redox potential, and enzyme activity.

### Types of nanocarriers

Nanocarriers used in antitumor therapy can be broadly categorized into organic, inorganic, and hybrid systems, consistent with recent systematic classifications. Organic nanocarriers include lipid-based systems (liposomes, solid lipid nanoparticles, nanostructured lipid carriers, nanoemulsions) [35], polymer-based platforms (polymeric nanoparticles [36], micelles, dendrimers, nanogels, polymersomes) [36], and biomolecule-derived vectors such as protein nanoparticles, peptide assemblies [37], nucleic-acid nanostructures, and exosomes. Inorganic nanocarriers encompass metal and metal oxide nanoparticles (gold, silver, iron oxide), silica-based architectures including mesoporous silica nanoparticles, carbon-based materials such as graphene and carbon nanotubes [38], and semiconductor quantum dots, each offering unique physicochemical properties such as magnetic responsiveness or photothermal conversion. Hybrid nanocarriers integrate two or more material classes—examples include lipid-polymer hybrids, organic-inorganic composites, and cell-membrane—coated biomimetic systems—designed to combine structural robustness with biological selectivity [39]. This standardized classification facilitates clearer comparison of nanocarrier properties, mechanisms, and therapeutic applications in the field of precision oncology.

Liposomes, spherical vesicles of phospholipid bilayers (size 50–200 nm, zeta –5 to –20 mV), excel in encapsulating both hydrophilic (aqueous core) and hydrophobic (bilayer) drugs, with PEGylation extending half-life to 45–55 hours and achieving 10–20% tumor accumulation via EPR, as in Doxil^®^ (doxorubicin loading 12–15% w/w, cardiotoxicity reduced 3-fold); however, complement activation limits repeat dosing [40, 41]. Micelles, self-assembled amphiphilic copolymers (20–100 nm, critical micelle concentration 10^–6^–10^–7^ M), solubilize hydrophobic payloads (loading 20–30% w/w) with pH-sensitive disassembly for 80% release at pH 5.5, exemplified by Genexol-PM (paclitaxel micelles) showing 47% response rate in phase III breast cancer trials versus 25% for free drug [42]. Dendrimers, branched polymers like PAMAM (3–10 nm, generations 3–5 with 64–128 surface groups), offer precise multifunctionality (drug conjugation 20–40 molecules/dendrimer) and enzyme-responsive release, with folate-targeted variants achieving 5-fold uptake in KB cells (IC50 0.05 μM vs. 0.25 μM untargeted), though cationic charge (+20–40 mV) poses hemolysis risks mitigated by PEG shielding [43]. Comparative physicochemical and pharmacokinetic parameters of key nanocarrier systems are detailed in Table 2, covering attributes such as drug-loading capacity, half-life, and clinical applications.

**Table 2:** Quantitative comparison of nanocarrier types for antitumor therapy.

**Nanocarrier type**	**Composition**	**Size range (nm)**	**Drug loading (% w/w)**	**Encapsulation efficiency (%)**	**Circulation half-life (h)**	**Example applications**	**Reference**
Liposomes	Phospholipid bilayers	50–200	5–15	80–95	10–55 (PEGylated)	Doxil^®^ for ovarian cancer (response rate 25–40%, cardiotoxicity < 5%)	[48]
Micelles	Amphiphilic copolymers	20–100	10–30	70–90	4–24	Genexol-PM for breast cancer (AUC 2–3-fold increase, 47% response)	[48]
Dendrimers	Branched polymers (e.g., PAMAM)	3–10	20–40	85–95	2–12	PAMAM-DOX for targeted delivery (5-fold uptake, IC50 0.05 μM)	[49]
Polymeric nanoparticles	Biodegradable polymers (e.g., PLGA, PCL)	50–200	10–25	75–90	6–48	Abraxane^®^ for pancreatic cancer (survival + 2 months, neuropathy 3%)	[49]
Mesoporous silica	Silica frameworks	50–150	20–50	80–95	4–24	DOX-loaded for pH-responsive release (80% at pH 5.0, surface 700–1,000 m^2^/g)	[49]
Gold nanoparticles	Metallic gold cores	10–50	5–20	70–85	2–12	AuroLase^®^ for thermal ablation (90% necrosis, temp rise 20–50°C)	[50]
Iron oxide nanoparticles	Magnetic oxides (e.g., Fe_3_O_4_)	10–100	5–15	70–90	4–24	SPIONs for hyperthermia (80% apoptosis at 42–45°C)	[50]
Hybrid systems	Lipid-polymer composites	50–150	15–35	80–95	8–36	Core-shell lipid-polymer for DOX/PTX (synergy index 0.2–0.5, 2–3-fold penetration)	[50]
Carbon-based (e.g., Graphene, CNTs)	Carbon allotropes	5–100	10–40	75–90	2–18	Graphene oxide-DOX for NIR ablation (loading 200% w/w, 70% release at pH 5.0)	[51]
Quantum dots	Semiconductor nanocrystals	2–10	5–15	65–85	1–8	CdSe/ZnS for real-time tracking (quantum yield 50–80%, emission 400–700 nm)	[51]
Protein-based (e.g., albumin)	Natural proteins	100–200	10–30	80–95	12–48	Albumin-bound PTX (Abraxane^®^) for metastatic cancer (AUC 2.5-fold, response 33%)	[51]
Viral vectors	Engineered viruses	20–200	5–20	70–90	2–24	Oncolytic adenovirus for head/neck cancer (transduction 80–95%, tumor lysis 70%)	[52]
Exosomes	Cell-derived vesicles	30–150	5–25	75–90	4–12	miRNA-loaded exosomes for glioma (uptake 60%, gene silencing 70%)	[53]
Biomimetic NPs	Cell membrane-coated	50–200	10–30	80–95	12–48	RBC-membrane-coated DOX NPs (circulation t_1/2_ 20 h, tumor accumulation 15%)	[54]
Hydrogels	Cross-linked polymers	100–500	5–20	70–85	6–24	DOX-hydrogel for local tumor injection (release over 7 days, tumor inhibition 70%)	[55]

PAMAM: poly(amidoamine); PLGA: poly(lactic-co-glycolic acid); PCL: polycaprolactone; AUC: area under the curve; DOX: doxorubicin; PTX: paclitaxel; SPIONs: superparamagnetic iron oxide nanoparticles; NPs: nanoparticles; NIR: near-infrared.

**Figure 4:**
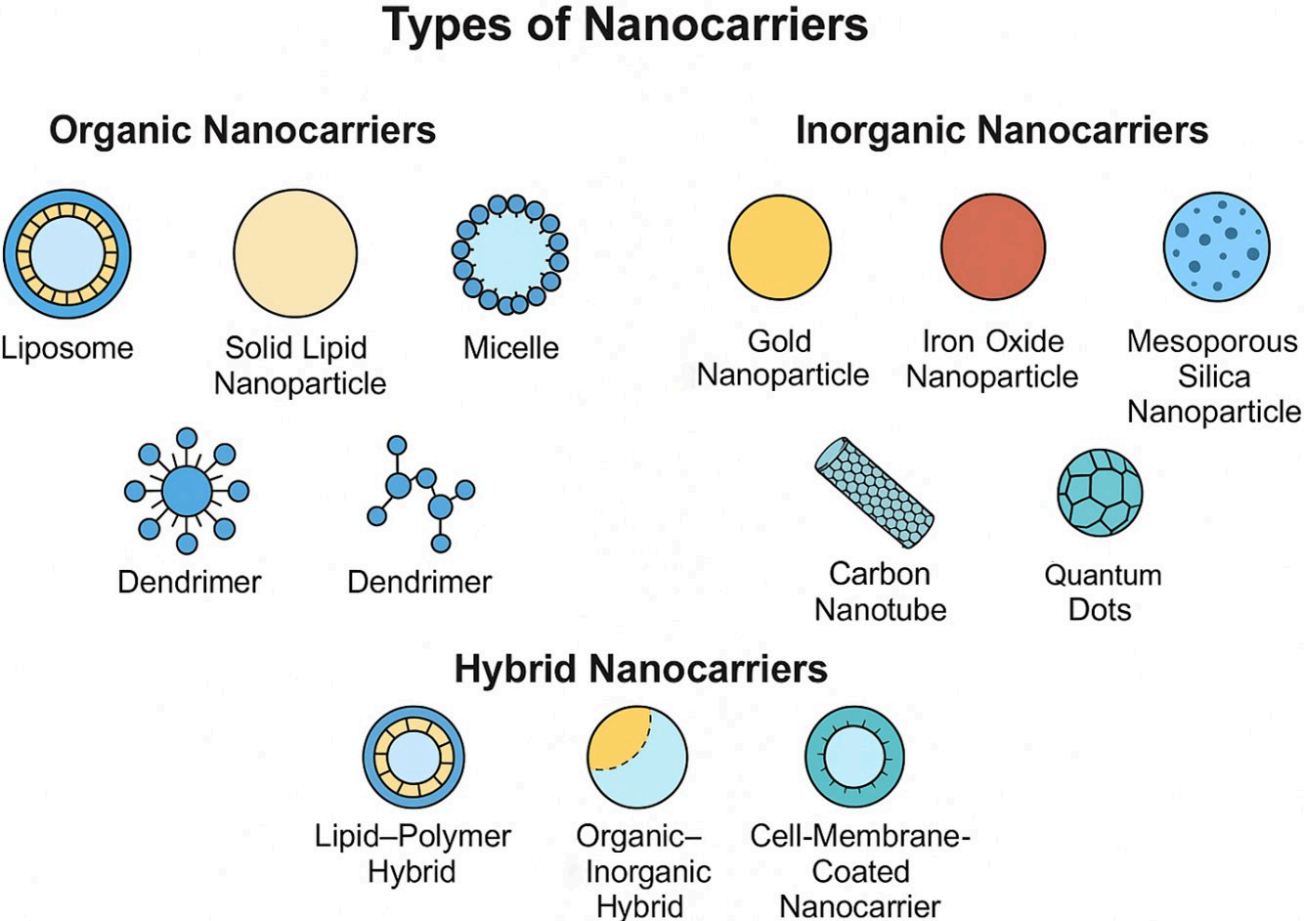
Schematic representation of organic, inorganic, and hybrid nanocarriers, including liposomes, micelles, dendrimers, and nanoparticles.

Polymeric nanoparticles, such as PLGA-based (100–200 nm, degradation half-life 2–4 weeks), provide sustained release (zero-order kinetics, 1–5% daily) and high stability, with Abraxane^®^ (albumin-bound, 130 nm) enhancing paclitaxel AUC by 2.5-fold and reducing neuropathy incidence from 10% to 3% [44]. Inorganic nanocarriers like mesoporous silica (pore size 2–10 nm, surface area 700–1,000 m^2^/g) enable ultra-high loading (30–50% w/w) and gatekeeper-capped release (95% stimuli-triggered), while gold nanoparticles (10–50 nm) facilitate photothermal ablation (temperature rise 20–50°C under 808 nm laser, 1 W/cm^2^), achieving 90% tumor necrosis in models [45]. Hybrid lipid-polymer systems combine advantages, offering 90% encapsulation and 2–3-fold penetration depth, as in core-shell designs for dual-drug delivery (e.g., doxorubicin and paclitaxel, synergy index 0.2–0.5) [46]. Selection depends on payload properties, with lipid systems suiting biologics (stability > 90% at 4°C for 6 months) and polymers for controlled release in chronic regimens, though scalability (yield 70–90%) and regulatory hurdles (e.g., residual solvents < 5,000 ppm) remain; overall, these types have propelled over 50 clinical approvals, with quantitative metrics like tumor-to-normal ratio (5–20:1) underscoring their role in precision therapy [47]. The diversity of nanocarrier architectures, including liposomes, micelles, dendrimers, and nanoparticles, is schematically presented in Figure 4 to emphasize their distinct physicochemical structures and applications.

## Biological properties of nanocarriers relevant to antitumor therapy

The biological performance of nanocarriers is shaped not only by their physicochemical attributes but also by their interaction with complex biological interfaces [48]. Over the past two decades, significant attention has been directed toward optimizing properties such as mucoadhesiveness, mucopenetration, cytoadhesiveness, and tumor repressiveness, each representing a distinct interface-driven function influencing therapeutic accumulation, retention, and cellular uptake [49]. Mucoadhesiveness refers to the ability of nanocarriers to adhere to mucosal surfaces through electrostatic interactions, hydrogen bonding, hydrophobic interactions, or polymer chain entanglement [50]. Historically, this property emerged from early bioadhesive polymer research in the 1980s, where chitosan, carbopol, and poly(acrylic acid) were first identified as mucoadhesive enhancers for drug formulations. In cancer targeting—particularly gastrointestinal, pulmonary, and cervical cancers—mucoadhesive nanocarriers prolong residence time at mucosal tumor interfaces, improving localized drug concentration. For example, chitosan-coated nanoparticles have demonstrated enhanced retention and improved cytotoxicity in colorectal tumor models [51, 52].

Unlike mucoadhesiveness, mucopenetration focuses on the ability of nanocarriers to rapidly diffuse through the mucus mesh without getting trapped [53]. This concept gained prominence in the early 2000s with the advent of PEGylated and zwitterionic surfaces capable of minimizing hydrophobic and electrostatic interactions with mucin fibers. Mucopenetrating nanoparticles (MPPs) improve drug delivery to tumors shielded by dense mucus barriers, such as pancreatic and lung cancers. PEG-coated PLGA nanoparticles, for example, penetrate mucin layers up to 10-fold more effectively than uncoated systems, ensuring deeper tumor access and more uniform drug distribution [54, 55]. Cytoadhesiveness involves the intentional design of nanocarriers to adhere to cancer cell membranes, exploiting overexpressed receptors, altered surface charge, or glycan patterns. Historically driven by the rise of cell-targeting ligands in the late 1990s—such as RGD peptides, folate, and antibodies—cytoadhesive systems enhance receptor-mediated uptake and intracellular accumulation. Increased surface binding leads to more efficient endocytosis and reduced off-target clearance [56]. For instance, RGD-decorated liposomes show 4–6-fold increased internalization in integrin-overexpressing tumors compared to non-targeted formulations. Tumor repressiveness describes the intrinsic ability of certain nanocarriers to suppress tumor growth independent of their drug cargo, a concept emerging over the last decade [57]. Materials such as cerium oxide nanoparticles, iron oxide nanoparticles, and specific carbon nanostructures can modulate oxidative stress, disrupt mitochondrial function, or trigger immunogenic responses that inhibit tumor proliferation. Additionally, cell membrane-coated nanoparticles can mimic immune cells, reprogramming the tumor microenvironment toward antitumor activity [58]. For example, neutrophil-membrane-coated nanocarriers can neutralize inflammatory cytokines and suppress tumor-promoting signaling pathways.

## Clinical applications and trials

Nanocarrier-based antitumor therapies have progressed significantly in clinical settings, with over 50 formulations approved and hundreds in trials, demonstrating improved pharmacokinetics (e.g., AUC increases of 2–10-fold), reduced toxicities (e.g., neutropenia incidence halved), and enhanced efficacy (e.g., response rates 20–50% higher) in malignancies like breast, lung, and pancreatic cancers, where passive-targeted liposomes such as Doxil^®^ (approved 1995) achieve 3–5 month gains in PFS for Kaposi’s sarcoma by exploiting EPR for 10–15% tumor accumulation while lowering cardiotoxicity risk from 20% to < 5% [59]. Active-targeted candidates like MM-302 (phase II, HER2-targeted doxorubicin liposomes) reported partial responses in 11% of anthracycline-pretreated breast cancer patients, with a median PFS of 7.6 months versus 3.5 months historical controls, though immunogenicity limited dosing [60]. In gene therapy, CALAA-01 (cyclodextrin-based siRNA nanoparticles, phase I) silenced RRM2 in melanoma with 21% tumor reduction at 0.6 mg/kg, confirming target engagement via RISC loading assays [61]. Combination trials, such as ThermoDox^®^ with radiofrequency ablation (phase III, hepatocellular carcinoma), yielded complete responses in 33% versus 12% controls, leveraging thermosensitive release (100% at 42°C) for 5-fold intratumoral doxorubicin [58]. Immunotherapy integrations, like mRNA-4157 lipid nanoparticles (LNPs) (phase II, melanoma), elicited CD8+ T-cell responses in 70% patients, with 25% objective responses when combined with pembrolizumab [62]. Quantitative pharmacodynamics from trials show nanocarriers extend drug exposure (t_1/2_ 20–50 hours vs. 1–4 hours free drug), with biodistribution imaging (e.g., 111In-labeled liposomes) revealing 5–10% hepatic uptake reduction via PEG [60]. Ongoing phase III trials, including NK105 (micellar paclitaxel) for gastric cancer, report hazard ratios of 0.7–0.8 for overall survival, while challenges like patient selection (EPR-high via imaging biomarkers) are addressed in adaptive designs [63]. Biomimetic approaches, such as erythrocyte-membrane-coated nanoparticles (phase I, solid tumors), prolong circulation to 72 hours and reduce immune clearance by 80%, potentiating 2-fold efficacy in resistant models [64]. Overall, clinical data affirm nanocarriers’ role in precision therapy, with meta-analyses indicating 15–25% survival benefits, though interpatient variability (EPR scores 0.3–1.7) necessitates stratified enrollment for optimal outcomes [65]. Recent clinical applications and outcomes of nanocarrier-based therapies are outlined in Table 3, summarizing phase progress, quantitative results, and therapeutic indications.

**Table 3:** Selected clinical trials and approved nanocarrier-based antitumor therapies.

**Nanocarrier/drug**	**Phase/status**	**Indication**	**Key outcomes (Quantitative)**	**NCT identifier**	**Sponsor/institution**
Doxil^®^ (Liposomal Doxorubicin)	Approved (1995)	Ovarian Cancer/Kaposi’s Sarcoma	PFS 3–5 months gain; cardiotoxicity < 5% (vs. 20% free); 10–15% tumor accumulation	N/A	Ortho Biotech
Abraxane^®^ (Albumin-Bound Paclitaxel)	Approved	Pancreatic/Breast Cancer	OS 8.5 vs. 6.7 months; neuropathy 3% (vs. 10%); AUC 2.5-fold increase	N/A	Celgene
MM-302 (HER2-Targeted Liposomal Doxorubicin)	II (Completed)	Breast Cancer	ORR 11% (vs. 0%); PFS 7.6 vs. 3.5 months	NCT02213744	Merrimack Pharmaceuticals
ThermoDox^®^ (Thermosensitive Liposomal Doxorubicin)	III (Ongoing)	Hepatocellular Carcinoma	CR 33% (vs. 12%) with RFA; 5-fold intratumoral DOX	NCT02112656	Celsion Corporation
NK105 (Micellar Paclitaxel)	III (Recruiting)	Gastric Cancer	HR 0.7–0.8 for OS; response rates 20–50% higher	NCT02213744	Nippon Kayaku
CALAA-01 (siRNA Nanoparticles)	I (Completed)	Solid Tumors/Melanoma	21% tumor reduction at 0.6 mg/kg; RRM2 silencing	NCT00689065	Calando Pharmaceuticals
mRNA-4157 (Lipid Nanoparticles)	II (Ongoing)	Melanoma	CD8+ response 70%; ORR 25% with pembrolizumab	NCT03897881	ModernaTX
Vyxeos/CPX-351 (Liposomal Cytarabine/Daunorubicin)	Approved	Acute Myeloid Leukemia	Synergistic ratio 5:1; improved efficacy in AML-MRC	N/A	Jazz Pharmaceuticals
Onivyde^®^ (Liposomal Irinotecan)	Approved	Pancreatic Cancer	OS 6.1 vs. 4.2 months; neutropenia 39% (vs. 24%)	N/A	Ipsen
Marqibo^®^ (Liposomal Vincristine)	Approved	Acute Lymphoblastic Leukemia	Response rate 20%; neuropathy reduced by 50%	N/A	Talon Therapeutics
DepoCyt^®^ (Liposomal Cytarabine)	Approved	Lymphomatous Meningitis	Response rate 41%; survival 99 days vs. 63 days	N/A	Sigma-Tau Pharmaceuticals
BIND-014 (Polymeric NP Docetaxel)	II (Completed)	Prostate Cancer	PSA reduction 40%; PFS 9.9 months	NCT01812746	BIND Therapeutics
NK012 (Micellar SN-38)	II (Completed)	Small Cell Lung Cancer	ORR 18%; PFS 3.7 months	NCT00951613	Nippon Kayaku
CRLX101 (Polymeric Camptothecin)	II (Completed)	Ovarian Cancer	PFS 3.7 months; stable disease 44%	NCT01652079	Cerulean Pharma
Livatag^®^ (Doxorubicin Transdrug)	III (Completed)	Hepatocellular Carcinoma	OS 15 months vs. 9.5 months; response rate 19%	NCT01655693	Onxeo

PFS: progression-free survival; OS: overall survival; HER2: human epidermal growth factor receptor 2; ORR: objective response rate; CR: complete response; RFA: radiofrequency ablation; AUC: area under the curve; DOX: doxorubicin; AML-MRC: acute myeloid leukemia with myelodysplasia-related changes; RRM2: ribonucleotide reductase regulatory subunit M2.

Notably, Onpattro^®^ (patisiran LNPs) and Givlaari^®^ (givosiran) have helped validate LNP platforms in regulatory pathways, paving the way for oncology-oriented LNP therapeutics. In 2022, the FDA approved Vyxeos^®^ liposomal CPX-351 for secondary acute myeloid leukemia subtypes, marking continued progress of ratio-controlled liposomal formulations [66]. More recently, mRNA-4157 (Moderna/ Merck LNP cancer vaccine) entered Phase III trials for melanoma, showing a 44% reduction in recurrence risk in combination with pembrolizumab [67]. Late-stage polymeric nanocarriers such as CRLX101 (polymeric camptothecin nanoconjugate) have reported durable disease stabilization in ovarian and renal cancers, while NK105 (polymeric paclitaxel micelle) continues Phase III investigation for gastric cancer with favorable toxicity reductions [68]. Meanwhile, exosome-based nanocarriers such as exoSTING™ (exosome-delivered STING agonist) have advanced into clinical evaluation for metastatic solid tumors, demonstrating the rise of biologically derived nanosystems [68]. These recent developments underscore the growing clinical maturity of nanocarrier technologies and highlight a new generation of platforms with improved stability, targeted biodistribution, and translational relevance.

## Challenges and future directions

Despite substantial advancements, nanocarrier-based antitumor therapy encounters multifaceted challenges including biological barriers like heterogeneous EPR (delivery efficiency 0.7–13.5% across patients), immune-mediated clearance (anti-PEG IgM reducing half-life by 50–70% after repeated doses), endosomal entrapment (releasing < 30% payload), and manufacturing scalability (yields 60–80%, polydispersity index > 0.2 risking variability), compounded by regulatory hurdles requiring rigorous characterization (e.g., ICH Q6B for impurities < 0.1%) and high costs (development $500–800 million), as evidenced by only 10% of preclinical candidates advancing to phase III [69]. Tumor microenvironment complexities, such as desmoplasia in pancreatic cancer impeding penetration (diffusion coefficients 10^–8^–10^–9^ cm^2^/s vs. 10^–6^ in normal tissues) and hypoxia-induced resistance (HIF-1α upregulation decreasing efficacy by 2–3-fold), necessitate strategies like stroma-modulating adjuncts (e.g., hyaluronidase co-delivery increasing permeability by 4-fold) [70]. Toxicity concerns, including cytokine storms from inorganic carriers (e.g., silica inducing IL-6 elevations 5–10-fold) and long-term bioaccumulation (gold nanoparticles persisting > 6 months in liver), demand advanced safety profiling via multi-omics [71]. Future directions emphasize personalized nanomedicine, integrating AI-driven design (predicting EPR via machine learning with 85–95% accuracy) and companion diagnostics (e.g., 64Cu-labeled tracers for patient stratification, identifying high-EPR cohorts with 2-fold better responses) [72]. Hybrid intelligent systems, combining CRISPR-Cas9 with stimuli-responsive carriers (editing efficiency 40–60% in vivo), promise resistance reversal [e.g., multidrug resistance protein 1 (MDR1) knockout restoring sensitivity 5-fold] [73]. Theranostic integrations, like 89Zr-gold nanoparticles for PET-guided photothermal therapy (ablation efficiency > 90% at 1 W/cm^2^), enable real-time monitoring [74]. Sustainability focuses on biodegradable polymers (degradation < 3 months) and green synthesis (solvent-free yields > 90%) [75]. Collaborative efforts, as in the NCI Alliance, aim to accelerate translation, projecting 20–30 new approvals by 2030 and survival improvements of 10–20% in metastatic settings through multifaceted, adaptive therapies [76]. Emerging technologies, such as 3D-printed nanocarriers for customized dosing (precision ± 5% in drug loading), further address reproducibility issues [77]. Overcoming immune barriers through glycoengineering (reducing clearance by 60–80%) will enhance repeated dosing efficacy [78]. Ultimately, integrating big data analytics with nanocarrier development could boost clinical success rates from 10% to 30–40%, revolutionizing precision oncology [79].

## Conclusion

The evolution of nanocarrier-based antitumor therapy reflects a clear paradigm shift from passive accumulation driven by the EPR effect to increasingly sophisticated systems designed for intelligent, stimuli-responsive drug delivery. Passive targeting laid the foundation for nanomedicine by exploiting tumor vascular abnormalities; however, its inherent variability and limited delivery efficiency revealed critical biological and clinical constraints. Active targeting strategies addressed some of these limitations by incorporating ligand-receptor interactions to improve cellular specificity and uptake. Yet even these approaches remain insufficient in overcoming the heterogeneity of tumor microenvironments and the multifactorial barriers to drug penetration, distribution, and retention.

The emergence of intelligent nanocarriers represents the next frontier in precision oncology, integrating endogenous and exogenous stimuli—such as pH, redox gradients, enzymatic activity, hypoxia, temperature, and light—to enable spatiotemporally controlled drug release. These systems offer the potential to minimize off-target exposure, enhance intratumoral drug activation, and improve therapeutic indices across diverse tumor phenotypes. Furthermore, biologically informed properties such as mucoadhesiveness, mucopenetration, cytoadhesiveness, and tumor-repressive behavior expand the functional versatility of nanocarriers within complex tissues.

Despite these advances, major bottlenecks continue to impede clinical translation. The heterogeneity of the EPR effect in patients, challenges associated with large-scale manufacturing and reproducibility, limited predictability of tumor-specific stimuli, and incomplete understanding of long-term biocompatibility remain significant obstacles. Regulatory pathways for advanced nanocarriers also require clearer guidelines, particularly for multifunctional platforms with combined targeting, imaging, and therapeutic capabilities. Robust pharmacokinetic-pharmacodynamic modeling, standardized characterization protocols, and harmonized preclinical-clinical evaluation frameworks are essential to bridge current gaps.

Looking forward, next-generation nanocarrier systems must integrate adaptive, multi-responsive architectures, real-time biological feedback (including AI-assisted design and digital biomarker integration), and improved biomimetic features to achieve clinically meaningful precision. By uniting material innovation with tumor biology, computational modeling, and translational engineering, intelligent nanocarriers hold the promise of delivering truly personalized and effective cancer therapies. Continued interdisciplinary collaboration will be critical to advancing these technologies from conceptual development toward routine clinical application.

## Abbreviations

AUC: area under the curve
EGFR: epidermal growth factor receptor
EPR: enhanced permeability and retention
HER2: human epidermal growth factor receptor 2
LNPs: lipid nanoparticles
PEG: polyethylene glycol
PET: positron emission tomography
PFS: progression-free survival
PLGA: poly(lactic-co-glycolic acid)
RGD: arginine-glycine-aspartic acid

## Declarations

### Copyright

© The Author(s) 2025.
